# Phenotype prediction based on genome-wide DNA methylation data

**DOI:** 10.1186/1471-2105-15-193

**Published:** 2014-06-17

**Authors:** Thomas Wilhelm

**Affiliations:** 1 Theoretical Systems Biology, Institute of Food Research, Norwich Research Park, Norwich NR4 7UA, UK

**Keywords:** Epigenetics, DNA methylation, Cancer, Feature selection, Machine learning, Classifier

## Abstract

**Background:**

DNA methylation (DNAm) has important regulatory roles in many biological processes and diseases. It is the only epigenetic mark with a clear mechanism of mitotic inheritance and the only one easily available on a genome scale. Aberrant cytosine-phosphate-guanine (CpG) methylation has been discussed in the context of disease aetiology, especially cancer. CpG hypermethylation of promoter regions is often associated with silencing of tumour suppressor genes and hypomethylation with activation of oncogenes.

Supervised principal component analysis (SPCA) is a popular machine learning method. However, in a recent application to phenotype prediction from DNAm data SPCA was inferior to the specific method EVORA.

**Results:**

We present Model-Selection-SPCA (MS-SPCA), an enhanced version of SPCA. MS-SPCA applies several models that perform well in the training data to the test data and selects the very best models for final prediction based on parameters of the test data.

We have applied MS-SPCA for phenotype prediction from genome-wide DNAm data. CpGs used for prediction are selected based on the quantification of three features of their methylation (average methylation difference, methylation variation difference and methylation-age-correlation). We analysed four independent case–control datasets that correspond to different stages of cervical cancer: (i) cases currently cytologically normal, but will later develop neoplastic transformations, (ii, iii) cases showing neoplastic transformations and (iv) cases with confirmed cancer. The first dataset was split into several smaller case–control datasets (samples either Human Papilloma Virus (HPV) positive or negative). We demonstrate that cytology normal HPV+ and HPV- samples contain DNAm patterns which are associated with later neoplastic transformations. We present evidence that DNAm patterns exist in cytology normal HPV- samples that (i) predispose to neoplastic transformations after HPV infection and (ii) predispose to HPV infection itself. MS-SPCA performs significantly better than EVORA.

**Conclusions:**

MS-SPCA can be applied to many classification problems. Additional improvements could include usage of more than one principal component (PC), with automatic selection of the optimal number of PCs. We expect that MS-SPCA will be useful for analysing recent larger DNAm data to predict future neoplastic transformations.

## Background

DNA methylation (DNAm) has important regulatory roles in many biological processes and diseases. It is the only epigenetic mark with a clear mechanism of mitotic inheritance [1] and the only one easily available on a genome scale for epigenome-wide association studies [2]. In vertebrates, the most common form of DNAm is 5-methylcytosine. It is associated with changes in DNA-protein interactions and gene expression. High methylation levels in cytosine-phosphate-guanine (CpG)-rich promoter regions are strongly associated with transcriptional repression [2]. Recent DNAm studies provided new etiological insights for human diseases [3]. Interestingly, epigenetic changes can be induced by diet, with implications for health [4] and obesity [5].

Although significant progress has been made during the last decades, cancer remains a global problem of rising importance [6]. On the other hand, modern omics technologies also enable new approaches for fighting the disease. ‘Epigenetic changes can be the earliest initiating factor in a human cancer’ [7]. DNAm markers for cancer detection have been found and potential DNAm driver events have been discussed [8]. The identification of DNAm biomarkers indicating the risk of later cancer onset is improving early diagnosis and therapy.

Based on the hypothesis that epigenetic variability may reflect differential exposure to genetic and environmental risk factors, it has been proposed that CpG sites with high inter-individual methylation variability might indicate risk of complex diseases [9]. The fact that such sites occur more frequently in the promoters of developmental genes [9] is consistent with this hypothesis, although sites with inter-individual variability are stable within individuals over many years [9].

Recently, a classifier based on DNAm data to predict the risk of neoplastic development (EVORA = epigenetic variable outliers for risk prediction analysis) has been proposed [10]. It considers two features of a CpG’s methylation, the level of methylation and methylation variability. The latter is the primary feature (CpG) selection criterion, the first few hundred most hypervariable CpGs (variability of methylation, quantified by Bartlett’s test, is higher in case samples) are considered as ‘risk CpGs’. The risk score of an individual sample is the fraction of ‘risk CpGs’ with a methylation level higher than a given cut-off. The optimal number of CpGs considered as well as the optimal cut-off is found by optimizing the Area Under the Curve (AUC) through internal cross-validation in the training data. EVORA was superior to the two tested popular classification algorithms PAMR [11] and SPCA [12] (in case of PAMR and SPCA feature selection was based on differential methylation statistics alone) [10]. However, it is known since the first DNAm measurements in human cancer that widespread DNA hypomethylation is involved [13], but EVORA, by considering hypermethylated and hypervariable CpGs alone, does not account for such sites. Indeed, promoter hypomethylation has been used as criterion for detecting novel oncogenes in cancer [14]. A refined version of EVORA was used to predict later neoplastic development [15] in women with normal cytology according to a cervical screening test [16]. This version considered a third feature of CpG methylation, age-correlation. A CpG was classified as ‘risk CpG’ only if its methylation is both more variable in cases and positively age-correlated [15]. The authors note that the uterine cervix is ‘currently the only human organ allowing relatively easy access to the cell of origin of the associated cancer well in advance of the first morphological signs of neoplastic transformation’.

Supervised principal component analysis (SPCA) [12] was developed for the prediction of tumour patients’ survival times based on gene expression data and was later successfully applied to other classification problems. A samples risk score corresponds to its score on the first principal component (PC) or the corresponding score from a linear combination of several PCs (weighted by singular values). Standard PCA is considered as an unsupervised technique. In SPCA the features used for PCA are specifically selected for best association with the phenotype of interest (in the training data). In the original SPCA paper, the Cox score (a measure of the correlation between a gene’s expression level and patient survival) was used as the association criterion [12] and only genes with a Cox score above a given threshold were considered for PCA (singular value decomposition). Again, the optimal threshold was obtained from internal cross-validation.

Here we present Model-Selection-SPCA (MS-SPCA), an enhanced version of SPCA. SPCA determines one model for the prediction of test data from optimisation within the training data. In contrast, MS-SPCA determines several models that perform well in the training data and selects specific ones for the prediction of test data, based on parameters of the test data. This is a natural extension of SPCA as it answers the question of how to deal with situations where several equally well performing models are found during the training data internal cross-validation. MS-SPCA applies all these models to the test data and identifies the most appropriate subset of these models for final prediction.

MS-SPCA was applied to analyse publicly available genome-wide DNAm data of cervical screening samples (27,578 CpGs corresponding to 14,495 genes, mostly in promoter regions [17]). The cases considered correspond to three different stages of cervical cancer development: (i) women with no cytological signs of neoplasia that have later developed neoplastic transformations (cervical intraepithelial neoplasia of grade 2 or higher, CIN2+) as determined from a subsequent screen after three years, (ii) neoplastic transformations (CIN2+, two independent case–control datasets), (iii) fully developed cancer.

We show that hyper- and hypomethylated, hyper- and hypovariable and positively and negatively age-correlated CpGs exist that are significantly associated with the phenotype. Significant CpGs of one dataset overlap significantly with significant CpGs of other datasets. Genes corresponding to significant CpGs are enriched with developmental genes (polycomb group targets, PCGTs [18]) and cervical cancer genes [19].

In contrast to EVORA, MS-SPCA uses the full non-binary methylation information as well as hyper- and hypomethylated sites. CpGs most associated with the phenotype in the training data are used for prediction. The corresponding ranked list of association strength is created by simultaneous consideration of three features of CpG methylation: average methylation difference, methylation variability difference (both between cases and controls) and age-correlation. We use a ranks-weighting scheme to account for different weighting of these features. Different weights and numbers of best ranking CpGs are tested by cross-validation in the training data. Therefor we have used the leave-one-out (LOO) method (other methods such as 5- or 10-fold cross-validation could be used instead).

We present results for all 21 possible test data predictions, using either cytology normal case–control data or CIN2+ case–control data for training. Cancer data are not used for training because the methylation patterns are very pronounced and would yield many thousands of perfectly predicting models in the training data. We also divide the cytology normal women dataset into several age-matched subsets (either completely HPV+ or HPV-), enabling another 50 predictions. Altogether, we performed 71 predictions in comparison to EVORA. MS-SPCA outperforms EVORA in nearly all cases. In most cases the difference is statistically significant.

## Results

We analysed six case–control datasets of genome-wide DNAm data (from four independent datasets, see Methods). Samples from women with normal cytology were used as control groups in all datasets. Table 1 shows the corresponding numbers of case and control samples.

**Table 1:** Datasets used

**GEO**	**Name**	**# Cases**	**# Controls**
GSE30758	Normal	75	77
GSE30758	Normal HPV+	44	48
GSE30758	Normal HPV-	31	29
GSE20080	CIN2+(a)	18	30
GSE37020	CIN2+(b)	24	24
GSE30759	Cancer	48	15

The four columns show the GEO [37] accession numbers, name of the datasets and the corresponding numbers of contained case and control samples.

### Significantly associated CpGs

We tested all CpGs in the six datasets for average methylation difference (*t*-test, Mann–Whitney *U* test), methylation variation difference (Bartlett’s and Levene’s test) and methylation-age-correlation. Table 2 shows the numbers of corresponding significant CpGs. Each test was done for all CpGs (first row within a table cell), or only hyper- (row 2) and hypo-CpGs (row 3) (see Methods, e.g. 80 significant hypovariable CpGs according to Levene’s test in the Cancer data). The higher number of significant hyper-CpGs may reflect the biased CpG choice of the Illumina 27K chip [10]. Future studies using less biased methods will clarify the relative importance of hyper- and hypomethylation. Table 2 shows that methylation patterns are more pronounced in more advanced stages of cancer. Most significant CpGs are found in the data where cases correspond to fully developed cancer, intermediate numbers are found in CIN2+ data and almost no significant CpGs are detected in the Normal data (cases still cytology normal, but will later develop transformations).

**Table 2:** Numbers of significant CpGs (q-value < 0.05) according to five different tests

	**Normal**	**Normal HPV+**	**Normal HPV-**	**CIN2+(a)**	**CIN2+(b)**	**Cancer**
t	0	0	0	389	233	14811
	0	0	0	452	100	10383(7)
	0	0	0	8	140	4753(1)
MWU	0	0	0	403	1008	16990
	0	0	0	408	372	11320(97)
	0	0	0	10	646	5885(46)
Bartlett	2830	1837	1204	3035	3444	12023
	1614	1154	748	2208	1948	12209
	1194	707	468	847	1489	414
Levene	0	0	0	241	2	5881
	0	1	0	326	4	7178
	0	0	0	0	0	80
Age-corr.	16	385	68	13	89	473
	16	330	75	19	52	525
	1	49	13	5	31	69

*t*-test, Mann–Whitney *U* test, Bartlett’s test, Levene’s test and test for methylation-age-correlation. The three rows per cell correspond to all, hyper- and hypo-CpGs. Numbers of most significant CpGs that are completely separating cases from controls are given in brackets.

There are many significant CpGs in the Normal data according to Bartlett’s test, but these CpGs are not significant according to a permutation test. Often there is just a single sample largely deviating from the others, so permuting phenotypes does almost not change the p-value (note the nearly identical number of cases and controls). Accordingly, after permuting phenotypes of the Normal data one gets about the same number of significant CpGs, according to Bartlett’s test. The other tests do not suffer from the problem, the results are always corresponding to permutation test results.

Although there are no statistically significant hyper- or hypomethylated CpGs in the Normal data, intersection of the most significant CpGs of these data with the most significant CpGs of the CIN2+ and Cancer data shows a significant overlap (Table 3), indicating that many important CpGs are hidden below the threshold for genome-wide significance. This corresponds to a similar finding in genome-wide association studies (GWAS) where typically only the minority of a phenotype’s heritability can be explained by the statistically significant single-nucleotide polymorphisms (SNPs) (~100). However, taking into account all ~1 m SNPs allows explanation of most of the heritability [20], ‘most of the genetic variance is simply hidden below the threshold for genome-wide significant associations’ [21]. Significant CpGs in the Normal HPV+ dataset overlap more to the corresponding CIN2+ and Cancer dataset CpGs than Normal HPV- CpGs. This could indicate that the potential transformation causing methylation patterns are more pronounced in the HPV+ dataset, but the number of HPV+ samples is also higher.

**Table 3:** Numbers of joint CpGs amongst the 500 most significant ones

	**Normal vs CIN2+a**	**Normal vs CIN2+b**	**Normal vs Cancer**	**Normal HPV+ vs N.HPV-**	**Normal HPV+ vs CIN2+a**	**Normal HPV+ vs CIN2+b**	**Normal HPV+ vs Cancer**	**Normal HPV- vs CIN2+a**	**Normal HPV- vs CIN2+b**	**Normal HPV- vs Cancer**	**CIN2+a vs CIN2+b**	**CIN2+a vs Cancer**	**CIN2+b vs Cancer**
t	**32**	10	**19**	15	**44**	12	**20**	9	12	14	16	**140**	**22**
	**39**	15	**28**	11	**55**	17	**35**	9	7	16	**32**	**190**	**26**
	**18**	11	**24**	6	**22**	9	**19**	7	15	17	10	**39**	**18**
MWU	17	10	16	8	**28**	10	**16**	7	9	15	**19**	**119**	**22**
	**25**	13	17	7	**42**	13	**29**	9	8	11	**29**	**171**	**23**
	12	10	**27**	7	**18**	8	**21**	7	9	13	11	**50**	16
Bartlett	**34**	**22**	**23**	**28**	**26**	**19**	**21**	**37**	**36**	**27**	**54**	**45**	**45**
	**75**	53	**37**	**32**	**71**	**53**	**26**	**39**	**39**	**25**	**98**	**62**	**57**
	**35**	12	16	**28**	**41**	15	**20**	**37**	10	15	**24**	**26**	**18**
Levene	**60**	**22**	**19**	12	**40**	17	**19**	12	6	8	**49**	**70**	17
	**90**	**41**	**32**	14	**59**	**30**	**29**	12	14	17	**68**	**79**	**34**
	11	2	9	9	**18**	16	16	14	6	14	17	13	7
Age-corr.	15	**32**	10	**107**	16	**30**	9	13	**18**	11	**19**	7	9
	12	**43**	8	**138**	12	**37**	10	10	**30**	5	**18**	8	15
	**32**	**38**	**22**	**49**	**29**	**30**	**23**	**29**	**29**	16	13	**19**	12

CpGs were ordered according to five different tests (*t*-test, Mann–Whitney *U* test, Bartlett’s test, Levene’s test and test for methylation-age-correlation) and the number of overlapping CpGs between the first 500 of two datasets determined. The three rows per cell correspond to all, hyper- and hypo-CpGs. Significant overlaps (p < 0.01) are shown in bold.

Tables 4 and 5 provide additional evidence that the most significant CpGs correspond to genes involved in cancer onset. For best ranking CpGs, we identified the corresponding genes and determined their overlap to 538 known cervical cancer genes [19] and 1,591 developmental genes (polycomb group targets PCGT) [18]. Table 4 shows that cervical cancer genes are enriched in nearly all high-ranking genes. Genes from the Normal data overlap as significantly as genes from CIN2+ and Cancer data. Hypo-CpG-genes overlap as well as hyper-CpG-genes. Table 5 shows that hyper-CpG-genes are highly enriched with PCGT genes [18], in contrast to hypo-CpG-genes.

**Table 4:** Numbers of genes overlapping to 538 known cervical cancer genes

	**Normal**	**Normal HPV+**	**Normal HPV-**	**CIN2+(a)**	**CIN2+(b)**	**Cancer**
t	**39**	**42**	**45**	**39**	**40**	31
	**43**	**42**	**38**	29	**40**	25
	**43**	**38**	**44**	**39**	**46**	**46**
MWU	37	38	**46**	35	**47**	32
	38	**41**	**42**	30	**41**	26
	**39**	**43**	**45**	**43**	**51**	**45**
Bartlett	38	**42**	**47**	36	**57**	**49**
	**43**	**40**	**48**	**37**	**47**	**46**
	**47**	**42**	36	**44**	**63**	**41**
Levene	37	**48**	**42**	**40**	**48**	**46**
	35	**46**	**43**	**39**	38	**46**
	35	**40**	**45**	**43**	**55**	38
Age-corr.	36	**46**	31	**40**	**40**	**50**
	**39**	32	34	**47**	**50**	**44**
	35	**47**	29	35	37	**38**

Genes corresponding to the 1000 most significant CpGs taken (five tests: *t*-test, Mann–Whitney *U* test, Bartlett’s test, Levene’s test and test for methylation-age-correlation, mean length of gene lists: 931). The three rows per cell correspond to all, hyper- and hypo-CpGs. Significant overlaps (p < 0.01) are shown in bold.

**Table 5:** Numbers of genes overlapping to 1,591 developmental genes

	**Normal**	**Normal HPV+**	**Normal HPV-**	**CIN2+(a)**	**CIN2+(b)**	**Cancer**
t	**126**	**112**	77	**217**	93	**138**
	**151**	**137**	81	**232**	82	**163**
	61	62	84	75	**100**	46
MWU	**113**	**110**	71	**200**	93	**126**
	**127**	**126**	91	**218**	76	**166**
	59	66	72	84	**122**	47
Bartlett	**142**	**156**	**160**	**194**	**185**	**222**
	**195**	**198**	**161**	**243**	**206**	**240**
	96	89	**113**	84	**100**	45
Levene	**127**	**139**	79	**224**	**133**	**264**
	**157**	**159**	96	**244**	**175**	**271**
	71	74	83	80	80	40
Age-corr.	**161**	**188**	**129**	**108**	**102**	**109**
	**206**	**219**	**171**	**118**	**126**	**98**
	71	86	62	81	59	**95**

Genes corresponding to the 1000 most significant CpGs taken (five tests: *t*-test, Mann–Whitney *U* test, Bartlett’s test, Levene’s test and test for methylation-age-correlation, mean length of gene lists: 931). The three rows per cell correspond to all, hyper- and hypo-CpGs. Significant overlaps (p < 0.01) are shown in bold.

### Motivation for Supervised PCA approach

Table 2 shows that no single CpG can differentiate between case and control samples in the three Normal datasets. However, the leading principal components (PCs) corresponding to the best ranking CpGs do separate cases from controls. Additional file 1: Figure S1 shows for the Normal dataset that the leading PCs do significantly (p < 10^-15^) differentiate, if the CpGs with the highest average methylation difference are used (CpG order according to t- or MWU-test).

### Prediction of future neoplastic transformation, CIN2+ and Cancer

#### Selecting the best models from cross-validation in training data

**Figure 1:**
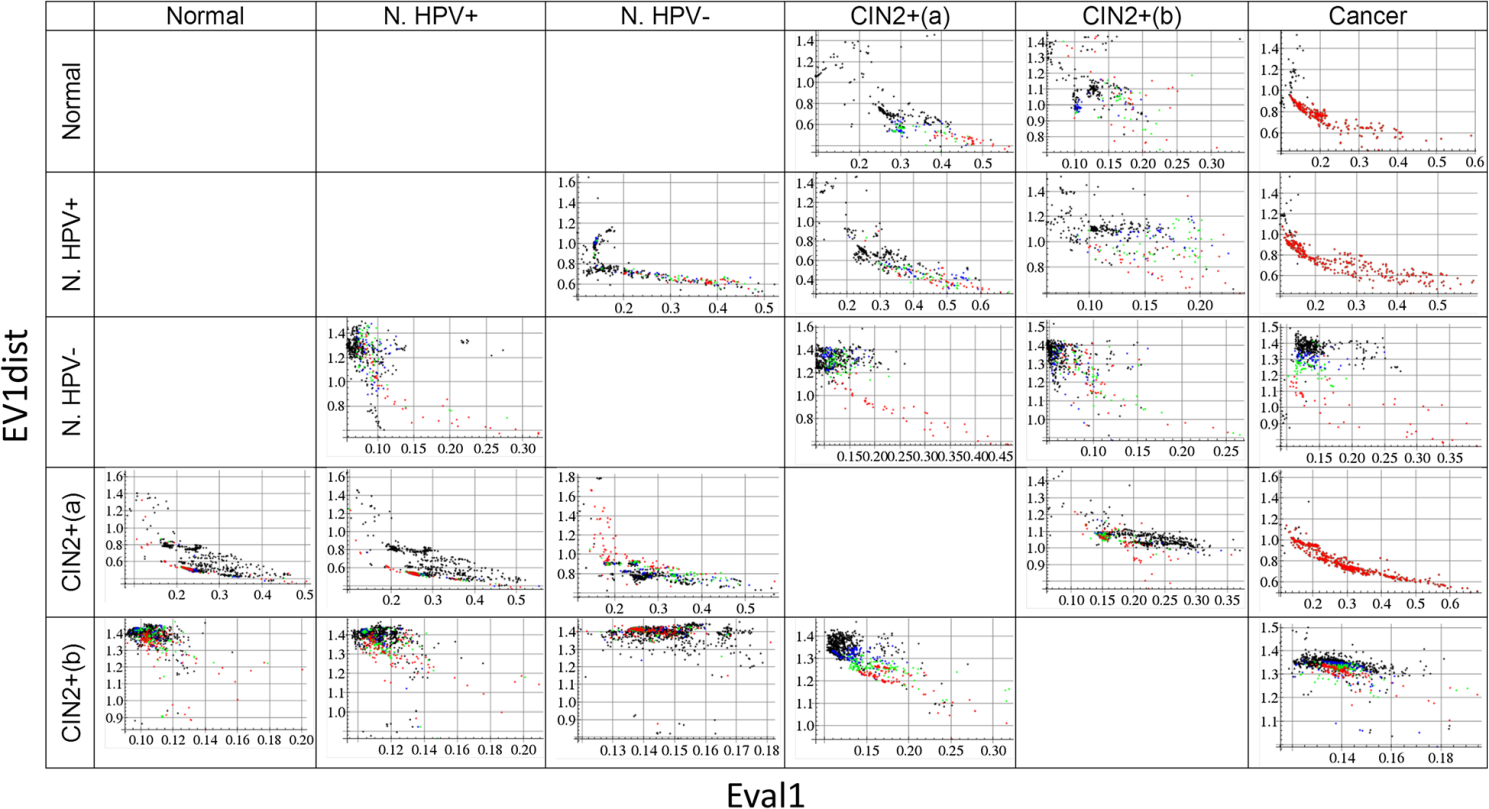
**Two parameters - used for final model selection.** Each dot corresponds to one model that performs well in cross-validation in the training data. Each row corresponds to a given training dataset (name on the left), each column to the corresponding test dataset (name in header). For instance, the field row 1 (Normal) – column 4 (CIN2+(a)) shows the two parameters (x-axis *Eval1*, y-axis *EV1dist*) for all >300 models selected from the training dataset Normal (LOO-prediction-accuracy > 0.65), when applied to the test data CIN2+(a). For better visualization, the 10% of the models predicting the test data best are shown in red, the next 10% (between deciles 1 and 2) are coloured green and the next (between deciles 2 and 3) blue. Black dots represent the remaining 70%. *Eval1* is the normalized largest eigenvalue of the covariance matrix taken from the methylation matrix of the test data. *EV1dist* is the Euclidean distance between the leading Eigenvectors of the model’s covariance matrix in the training data and in the test data.

**Figure 2:**
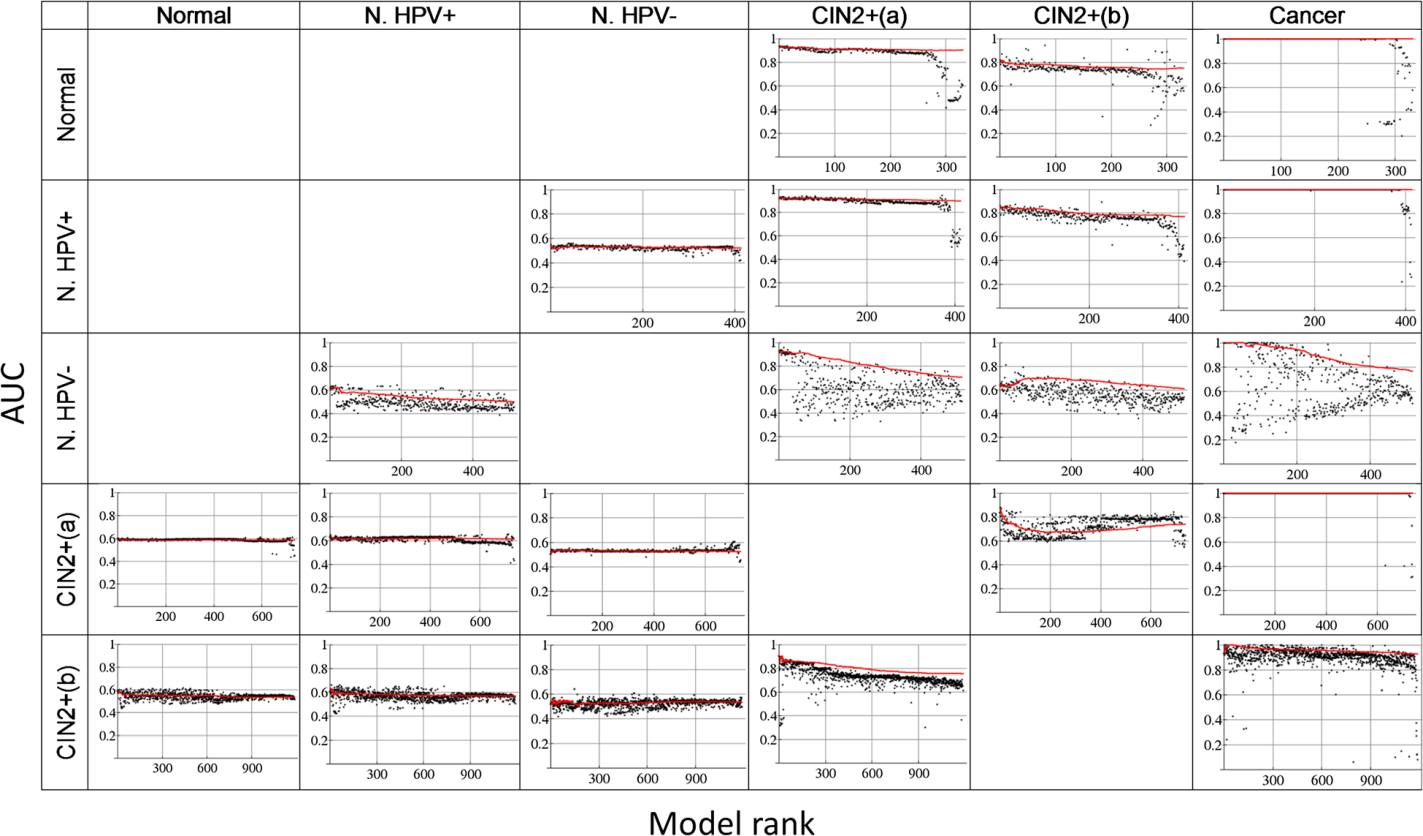
**Performance of prediction (AUC).** Each row corresponds to a given training dataset, each column to a test dataset and each dot to one model. Models are ordered according to *Eval1*-*EV1dist*, rank 1 corresponds to the model with the largest value. *Eval1* is the normalized largest eigenvalue of the covariance matrix taken from the methylation matrix of the test data. *EV1dist* is the Euclidean distance between the leading Eigenvectors of the model’s covariance matrix in the training data and in the test data. The red line shows the AUC resulting from cumulative risk scores (see Methods). The values of the red lines at model rank 5 are given in Table 6.

**Figure 3:**
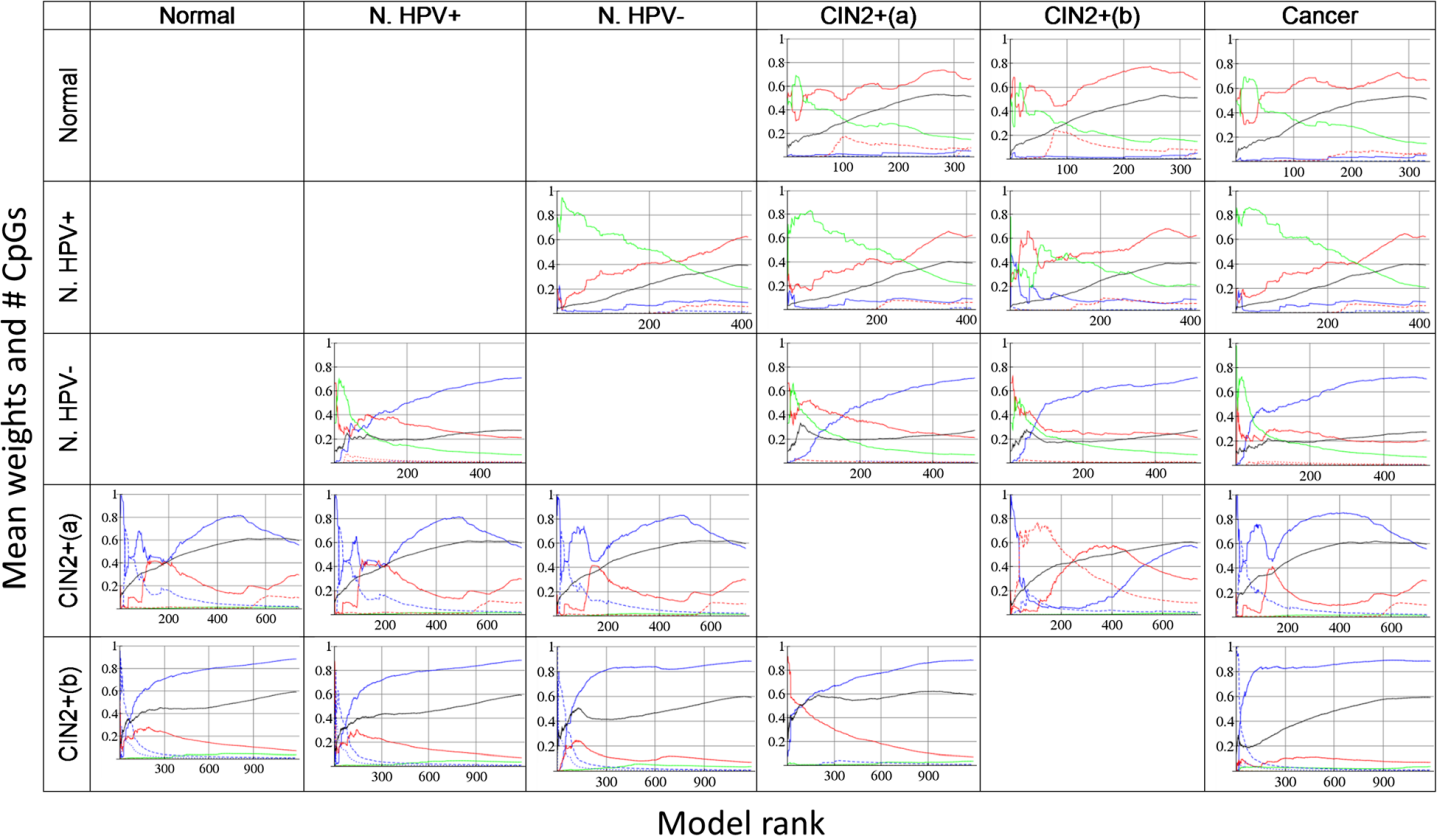
**Description of models used for predictions (weights and # CpGs).** Each row corresponds to a given training dataset, each column to a test dataset. Models are ordered according to *Eval1*-*EV1dist*, rank 1 corresponds to the model with the largest value. *Eval1* is the normalized largest eigenvalue of the covariance matrix taken from the methylation matrix of the test data. *EV1dist* is the Euclidean distance between the leading Eigenvectors of the model’s covariance matrix in the training data and in the test data. The black line shows the mean number of CpGs used in the models up to the indicated rank, normalized by the maximum number of CpGs considered (1500). The other lines correspond to the mean weights (see Methods) used in the models up to the indicated rank. Blue lines correspond to average methylation difference (t- or MWU test), red to methylation variation difference (Bartlett’s or Levene’s test) and green to methylation-age-correlation. Solid lines indicate models taking into account both hyper- and hypomethylated CpGs. Dashed lines represent models using only hypermethylated and dotted lines indicate models using only hypomethylated CpGs.

We tested models based on the best ranking CpGs according to CpG ‘combi’ orders (combining three features of a CpG’s methylation: average methylation difference, methylation variation difference and age-correlation; see Methods) for cross-validation prediction in the five datasets that served as training data: Normal, Normal HPV+, Normal HPV-, CIN2+(a) and CIN2+(b). In each case the first few hundred best performing models were applied for prediction of all the corresponding independent datasets (Table 6, Figures 1, 2 and 3). Models with the following cross-validation prediction accuracies were used: >0.65 for the three Normal datasets, >0.82 for CIN2+(a), and >0.95 for CIN2+(b); resulting in >300 models trained on the Normal datasets and >700 and >1000 models trained on CIN2+(a) and CIN2+(b), respectively (Figures 2 and 3). The models predicting the training-data best have the following accuracies: ~0.8 for the Normal datasets, 0.92 for CIN2+(a) and 1 for CIN2+(b) (~200 models with accuracy 1). The clearer patterns in CIN2+(b), compared to CIN2+(a), might result from the fact that all CIN2+(b) samples were taken from HPV infected women.

**Table 6:** Prediction performance (AUC) of MS-SPCA

	**Normal**	**Normal HPV+**	**Normal HPV-**	**CIN2+(a)**	**CIN2+(b)**	**Cancer**
Normal				**0.93**/0.66(0.87)	**0.81**/0.55(0.69)	**1**/0.81(0.94)
Normal HPV+			**0.52**/0.45	**0.93**/0.77	**0.84**/0.65	**1**/0.75
Normal HPV-		**0.61**/0.55		**0.92**/0.68	**0.64**/0.46	**1**/0.71
CIN2+(a)	**0.60**/0.57	**0.63**/0.60	0.53/**0.54**		**0.83**/0.71	**1**/0.72
CIN2+(b)	**0.58**/0.56	**0.62**/0.60	**0.53**/0.46	**0.87**/0.82**(0.87)**		0.98/0.85**(1)**

Rows correspond to training data and columns to test data. The first number shows the performance of MS-SPCA, the second the performance of EVORA (mean value of 8 runs). Numbers in brackets show the five EVORA results as presented in [10]. Bold numbers show best predictions.

#### Identifying the likely best predicting models and final prediction

MS-SPCA selects the final models for prediction on the basis of parameters obtained from analysis of the test data. Figure 1 depicts the two parameters *Eval1* and *EV1dist* for all used models. Figure 2 shows the prediction performance of all models ordered according to the difference *Eval1*-*EV1dist* (using standardised numbers for *Eval1* and *EV1dist*). *Eval1* is the normalized largest eigenvalue of the covariance matrix taken from the methylation matrix of the test data. *EV1dist* is the Euclidean distance between the leading Eigenvectors of the model’s covariance matrix in the training data and in the test data. It can be seen from the colouring in Figure 1 that the smaller *EV1dist* and the larger *Eval1* is, the more likely the model makes a good prediction of the test data. Red dots tend to be in the lower right corner, especially for the well predicted advanced transformation stages CIN2+ and cancer (least for the poorly predicted Normal HPV- samples). *Eval1* measures how much of the variation in the test data is likely captured by PC1. *EV1dist* is a measure for how well the model obtained from the training data fits to the test data. Importantly, the two parameters *Eval1* and *EV1dist* capture information about how likely a model makes a good prediction. For example, in the first row of Figures 1, 2 and 3 models were trained on the Normal dataset. These models predict the CIN2+(a) and Cancer data very well (Figure 2), but less so the CIN2+(b) data. Figure 1 shows that *Eval1* is generally smaller for CIN2+(b) models and *EV1dist* is larger. Similarly in the last column: in rows 1, 2 and 4, where the prediction is very good (Figure 2), *Eval1* is often large and *EV1dist* is small. Parameters such as *Eval1* and *EV1dist* open the possibility to automatically select the most appropriate number of principal components to consider for prediction. The authors of the original SPCA paper had worked on a similar problem but had not solved it [12]. Given that the models trained on CIN2+(b) data have consistently poor *Eval1* and *EV1dist*, compared to models from other training data, it seems that consideration of more than one PC could improve the prediction performance.

Although the two parameters *Eval1* and *EV1dist* are correlated to each other they also carry important unique information. Each parameter alone is inferior to the sum *Eval1*-*EV1dist*. For example, row 3-column 2 shows poorly performing models with small *EV1dist*, row 4-column 5 shows poor models with large *Eval1*.

Figure 2 shows that, although there is some variation, the best-ranking models (low ranks) perform better. Moreover, the cumulative score (see Methods) enables robust and good prediction by considering only best-ranking models. The final prediction results that correspond to the AUC of the cumulative score at model rank 5 are given in Table 6. The prediction of a given test dataset by MS-SPCA is quite consistent, no matter which training dataset is used: Normal ~0.6, Normal HPV+ >0.6, Normal HPV- >0.5, CIN2+(a) >0.9, CIN2+(b) >0.8, Cancer ~1.

Comparison with EVORA [10] was performed, using the R-package ‘evora’. Table 6 shows the mean prediction values of 8 independent runs. Prediction with MS-SPCA is nearly always superior. The better performance of MS-SPCA is statistically significant. A two-way ANOVA confirms the significantly higher performance of MS-SPCA, even in comparison to the three previously published prediction results of row 1 (using Normal for training): we simulated 20 EVORA-AUC values for each prediction such that the mean AUC and the 95% CI correspond to the numbers given [10] and compared to the AUCs of the top 20 ranking MS-SPCA models, p < 0.001. It can be seen that the numbers reported by the EVORA authors [10] are better than those from our own runs. The original EVORA method [10] and the ‘evora’ R-package do not consider any information about the age of the women who have provided the samples, although this was considered in a related paper [15]. It is possible that the higher numbers result from taking age into account. However, MS-SPCA outperforms these results as well. Moreover, MS-SPCA was also performing well without the use of age information. The results are similar to the ones presented in Table 6, mostly lower by about 0.01-0.02, but higher in the case of Normal HPV- → CIN2+(b) by 0.04.

#### Description of best performing models

A concise description of the models used for predictions is shown in Figure 3. Models were sorted according to *Eval1*-*EV1dist* and mean values were calculated from model rank 1 up to any other indicated rank. For instance, rank 5 shows the mean numbers of the first 5 models. Accordingly, the final rank shows the mean values of all the models used. Interestingly, the figures in a given row are similar, indicating that the model order is also similar for different test data predictions. Best-ranking models typically contain fewer CpGs (black line). Clearly, solid lines are dominating amongst the coloured ones. That means most of these models include hyper- and hypomethylated CpGs, models with pure hyper- or hypo-CpGs are rare. The models selected from cross-validation within Normal and Normal HPV+ data contain CpGs which are mostly ordered by methylation variation difference, the red line ends at >60% (~2/3 correspond to Bartlett’s test ordering, 1/3 to Levene’s test). Models in rows 3–5 are dominated by methylation difference ordering (>1/2 *t*-test in rows 3 and 5, ~2/3 MWU in row 4).

The best performing models (low ranks) are dominated by age-correlation in the Normal training data (rows 1–3) and by methylation difference in row 4. Hypermethylation and methylation variation difference (the features specifically selected by EVORA [10]) are dominating in the best predicting models in row 5.

#### Cytologically normal samples contain DNAm patterns predisposing for later transformations

Samples classified as normal by the standard cytological screening method contain DNAm patterns that can clearly differentiate between normal and CIN2+ samples and between normal and cancer samples (Figure 2 and Table 6). Interestingly, this conclusion even holds for the Normal HPV- samples. Although HPV infection is considered a necessary condition for neoplastic transformation and later cancer, the differentiating DNAm patterns exist prior to any HPV infection. These patterns might contribute to infection itself and/or contribute to transformations after infection. The latter possibility is supported by our finding that MS-SPCA trained on Normal HPV- data predicts the phenotype of Normal HPV+ samples about as well as when trained on CIN2+ data.

To further study the problem of phenotype prediction within the Normal data, a corresponding stratified 5-fold cross-validation was performed. Both the Normal HPV+ and the Normal HPV- datasets were subdivided into five equally sized case–control parts, always ensuring that age is matched. Five predictions were performed, taking one set for testing and the remaining four as training data. Additional file 1: Figures S2-S4 show the plots corresponding to Figures 1, 2 and 3 for the five cases using Normal HPV+ data for training, and Additional file 1: Figures S5-S7 the same using Normal HPV- for training. Clearly, *Eval1*-*EV1dist* is an important sorting criterion, for instance in cancer prediction from Normal HPV- data. However, Additional file 1: Figures S3 and S6 also show that this sorting is selecting the best predicting models in the test data corresponding to the training data (first column) less robustly. Our hypothesis that this is caused by the small size of these test sets (<10 cases and controls) was confirmed by a corresponding calculation (Additional file 1: Figure S8). The larger the test sets are the better *Eval1*-*EV1dist* selects the best predicting models. This implies that MS-SPCA would predict future transformations better in larger test data. Table 7 shows the results of the final MS-SPCA prediction using the cumulative score of the first 5 models, in comparison to EVORA predictions. MS-SPCA prediction results are better and more consistent (less variable) than EVORA results. Again, in most cases the advantage of MS-SPCA is statistically significant. MS-SPCA allows the prediction of later neoplastic transformation in HPV infected women with cytologically normal samples (mean AUC = 0.60).

**Table 7:** Prediction performance (AUC) of MS-SPCA, using Normal data for training

	**Normal HPV+ test1-5**	**Normal HPV+**	**Normal HPV-**	**CIN2+(a)**	**CIN2+(b)**	**Cancer**
N.HPV+ 1	**0.59**/0.39		**0.50**/0.38	**0.91**/0.78	**0.86**/0.65	**1**/0.73
N.HPV+ 2	**0.75**/0.50		**0.52**/0.41	**0.93**/0.69	**0.76**/0.53	**1**/0.52
N.HPV+ 3	**0.62**/0.54		**0.54**/0.49	**0.93**/0.84	**0.85**/0.70	**1**/0.70
N.HPV+ 4	0.47/**0.52**		0.50/**0.56**	**0.93**/0.74	**0.84**/0.64	**1**/0.79
N.HPV+ 5	**0.55**/0.53		0.51/0.51	**0.92**/0.74	**0.84**/0.58	**1**/0.68
	**Normal HPV- test1-5**					
N.HPV-1	**0.63**/0.31	**0.62**/0.53		**0.91**/0.69	**0.71**/0.55	**1**/0.80
N.HPV-2	0.14/**0.47**	**0.63**/0.59		**0.91**/0.67	**0.71**/0.57	**1**/0.76
N.HPV-3	0.42/**0.53**	**0.61**/0.60		**0.92**/0.67	**0.67**/0.56	**1**/0.72
N.HPV-4	0.36/**0.41**	**0.63**/0.59		**0.92**/0.67	**0.67**/0.56	**1**/0.76
N.HPV-5	0.39/**0.55**	**0.50**/0.47		**0.82**/0.51	**0.50**/0.39	**1**/0.67

Rows correspond to training datasets and columns to test datasets. The first number shows the performance of MS-SPCA, the second the performance of EVORA (mean value of 8 runs). Bold numbers show best predictions.

Finally, we also tested if DNAm patterns might contribute to infection itself by using the models trained on Normal HPV- data to predict independent test data where the “cases” are healthy HPV+ samples (women remain cytology negative for 3 years) and controls are healthy HPV- samples. Interestingly, this ‘phenotype’ can be predicted even better than the future transformation in the Normal HPV+ data: AUC > 0.8 (Additional file 1: Figure S9). This indicates that DNAm patterns may contribute to HPV infection.

### Consistency of models used for predictions, corresponding outstanding CpGs and genes

All phenotype predictions were based on the five models with the largest parameter *Eval1*-*EV1dist* (see Methods). Interestingly, the five models chosen for the prediction of different test data were often identical. For example, three of the five models from the training data Normal that were used to predict CIN2+(a), CIN2+(b) and Cancer (first row in Figures 1, 2 and 3) are identical, i.e. these three models predict the three different test data very well. Similar model consistency was also found using other data for training: Normal HPV+ and HPV- were used to predict four independent test data, CIN2+(a) and CIN2+(b) to predict five test data (cf. Table 6). The corresponding intersection of the 4x5 or 5x5 models is 1, 3, 3 and 0, respectively. Although there is no model amongst the five best that is used for all five test data predictions in the case of training data CIN2+(b), one model is consistently used to predict the four test data Normal, Normal HPV+, CIN2+(a) and Cancer; three of the models used to predict Normal HPV- were also used to predict Cancer.

Not surprisingly, the CpGs used in the five models for final prediction are also largely overlapping. Furthermore, the CpGs of all the five models used to predict one test dataset tend to overlap with the corresponding CpGs used to predict another test dataset. The numbers of common CpGs used for the prediction of all test datasets based on the five training datasets Normal, Normal HPV+, Normal HPV-, CIN2+(a) and CIN2+(b)) are 235, 75, 171, 238 and 89, respectively. For instance, 235 same CpGs were used to predict CIN2+(a), CIN2+(b) and Cancer from the training dataset Normal. There is one CpG occurring in all these five CpG lists: cg11965370, located in a CpG island 234 bp upstream of the transcription start site (TSS) of the NTM gene. NTM codes for the protein neurotrimin, which is known to play a role in cell-adhesion [22]. The intersection of the 235, 238 and 89 CpGs is cg00027083, cg11965370, cg16638540, cg22415432, cg22881914, cg25044651, cg26186727 and cg26363196, which are all hypermethylated in all six datasets.

Although there is no CpG with significant methylation difference between cases and controls in the three Normal data (Table 2), there are CpGs consistently used for the prediction of test data. The intersection of the 235, 75 and 171 CpGs is cg02008154, cg02250594, cg02624705, cg06277657, cg11965370, cg12457773, cg13870866, cg17727529, cg17861230, cg23303408, cg23316360, cg23710218, cg26963271, nearly all are hypermethylated in all six datasets.

139 CpGs occur in at least two of the five CpG lists with 130 of them being located in CpG islands, mostly close to the TSS (Additional file 2: 139CpGs.xlsx). Nearly all are hypermethylated in the analysed six datasets, but two are clearly hypomethylated: cg07251788 in all six datasets, cg08214029 in five datasets. They correspond to the genes CCL18 and CLTCL1, respectively. Both are often overexpressed in cancer cells. Elevated CCL18 expression plays a role in ovarian carcinoma [23] and induces metastasis of breast cancer [24]. CLTCL1 is overexpressed in cervical and other cancers [25-27]. Two genes correspond to more than two of the 139 CpGs, both are well-known tumour suppressor genes: DCC (DCC = Deleted in Colorectal Cancer) and GATA4. The GATA4 promoter is hypermethylated in cancer [28] and it is involved in ovarian cancer [29-31]. Interestingly, the two most important human DNA regions of a recent DNAm cervical pre-cancer classifier, EPB41L3 and DPYS [32], are amongst the ~100 regions corresponding to the 139 CpGs.

## Discussion

We have developed MS-SPCA, an advanced version of the classifier SPCA [12]. In contrast to SPCA, MS-SPCA considers several models that perform well in training data cross-validation and selects the final ones for prediction of test data, based on parameters obtained from the test data. We tested different parameters and parameter combinations based on the training data but none was performing as well in selecting the best models as our final criterion *Eval1-EV1dist*.

Importantly, the values of the parameters *Eval1* and *EV1dist* are related to the prediction performance. For instance, Figure 1 shows that, using CIN2+(b) for training, all corresponding models have relatively poor parameters *Eval1* and *EV1dist*, compared to the cases using datasets Normal or CIN2+(a) for training. Accordingly, the prediction performance using the CIN2+(b) dataset for training is lower (Figure 2). Consideration of >1 PC might improve the prediction. It seems that parameters such as *Eval1* and *EV1dist* could provide the basis for automatic selection of the most appropriate number of principal components to consider. We also tested a support vector machine (SVM) for prediction using more than one PC, but without any specific criterion for model selection. The corresponding performance was slightly lower, but a combination of an automatic selection of the number of PCs to consider with advanced learning algorithms such as SVM could lead to further improvement.

Another point for potential future improvement concerns the number of models to consider. Here we used the few hundred best models according to training data cross-validation performance. Obviously, some diversity of the models is needed such that the final model selection criterion *Eval1-EV1dist* can select the most appropriate ones. Considering only the very few in the training data top performing models might suffer from too low model diversity and over-fitting to the training data, considering many models is time consuming and might result in over-fitting to the test data. Maybe some optimal diversity (in terms of different weights and number of CpGs and/or different CpGs contained in the models) can be defined, helping to automatically select the cross-validation performance cut-off and therefore the number of models to consider.

We have shown that the model selection of MS-SPCA works better the more samples the test set contains. In the special case that the test set contains only one sample no test data covariance matrix can be calculated. The corresponding phenotype could be predicted by either a majority vote of all the models (selected from training data cross-validation) and/or by assigning additional samples to the test set (for instance taking some from the initial training set, but not using them for training) and applying the criterion *Eval1-EV1dist.*

MS-SPCA was applied for phenotype prediction from genome-wide DNA methylation data. The procedure involves the following steps: 1) splitting of the training data into k-fold training and test sets (here we used the LOO method), 2) calculation of p-values for all CpGs (in each of the k training datasets) for the three features of a CpG’s methylation: average methylation difference, methylation variation difference and methylation-age-correlation, 3) identification of the best models in training data cross-validation: testing models with different weights (combining the three p-value rank lists) and numbers of CpGs in each of the k training datasets 4) predicting the independent test data with the best performing models identified in step 3 (here we used the few hundred best models), 5) the Model Selection step of MS-SPCA: ranking these models according to the criterion *Eval1-EV1dist* and using the cumulative risk scores of the first n best ranking models for final prediction (here we used n = 5)*.* We have shown that MS-SPCA performs better than the recent method EVORA [10].

The first experiments on DNA methylation in human cancer showed widespread hypomethylation [13], but after the discovery of tumour suppressor genes one also observed promoter hypermethylation [33,34]. Meanwhile, of all epigenetic modifications, hypermethylation of promoter regions of tumor suppressor genes has been most extensively studied [35]. We have shown that hypermethylation and hypervariability are important factors for neoplastic transformation, but hypomethylation and hypovariability play a role as well. The 27K CpGs analysed here are biased towards hypermethylated promoter regions [10]. Nevertheless, MS-SPCA performs best if both hyper- and hypomethylated CpGs are considered. It will be interesting to see if hypomethylated CpGs play an even bigger role in more recent larger and less biased DNA methylation assays.

It has been suggested that hypervariable CpGs are most important for the prediction of early cancer development [15], although analysis of other cancer data does not always benefit from considering differential variability [10]. This is in agreement with our results (Figure 3). MS-SPCA in combination with the here presented CpG selection method allows for automatic identification of the most appropriate weighting of the CpG’s methylation features.

Although HPV infection is considered necessary for cervical cancer development [36], we have shown that specific DNA methylation patterns which are likely contributing to later cancer development exist already before any HPV infection. This is in accordance with previous findings that epigenetic changes may be the earliest initiating factor in a human cancer [7]. On the other hand, it was recently suggested that the earliest changes leading to later transformations might be other epigenetic changes such as chromatin modification rather than DNA methylation [33]. If this is true, then the patterns observed in the Normal data may be the consequence of other earlier epigenetic changes. However, even then the analysis of DNA methylation can be important for early diagnosis of dangerous transformations as it is detectable before standard cytological screening methods can show evidence of transformations [16].

We have presented evidence that DNA methylation patterns exist in cytology normal HPV- samples that (i) predispose to neoplastic transformations after HPV infection and (ii) predispose to HPV infection itself. It will be interesting to test if other virus infections are also correlated with specific DNA methylation patterns.

Given that MS-SPCA performs well using data from comparatively few samples and only 0.1% of the GpGs in the human genome [2], it is possible that future DNA methylation analysis can provide the accuracy needed for clinical applications.

## Conclusions

MS-SPCA, an advanced version of the classifier SPCA, is presented. It performs well in predicting phenotypes from genome-wide DNA methylation data. MS-SPCA can be applied to other classification problems. Consideration of more principal components, based on the automatic selection of the corresponding most appropriate number, and additional model selection methods might allow further improvement.

## Methods

### Data

Four independent age-matched case–control datasets were analysed, all freely available from the Gene Expression Omnibus repository [37], accession numbers GSE20080, GSE30760 (SuperSeries comprising GSE30758 and GSE30759) and GSE37020. CpG methylation was measured using Illumina’s Infinium Human Methylation 27K Beadchips [38] and corresponding β-values were calculated for 27,578 CpGs: β = M/(U + M + e), where U and M are the unmethylated and methylated intensity values of the CpG and e is a small correction term [10]. The four datasets are labeled according to the cancer stage of case samples (Normal, CIN2+(a), CIN2+(b) and Cancer): (1) Normal: 152 samples in a prospective case–control study (matched for human papillomavirus (HPV) status and age) - 75 women with normal cytology in screening round 1 but demonstrated CIN2+ after 3 years in round 2 (=cases) and 77 control women with normal cytology in rounds 1 and 2, (2) CIN2+(a): 48 samples (age-matched) with 18 cases (CIN2+, all HPV+) and 30 controls (normal cytology, HPV+ and HPV-), (3) CIN2+(b): 48 samples (all HPV+ and age-matched) with 24 cases (CIN2+) and 24 controls, (4) Cancer: 63 samples (age-matched) with 48 cases (cervical cancer) and 15 controls (no HPV information). Additionally, the 152 Normal samples were split into two datasets: 92 HPV+ (44 cases, 48 controls) and 60 HPV- (31 cases, 29 controls), yielding together six datasets (Table 1).

### Quality control and normalization

The six datasets were processed by the following procedure: Missing values were replaced by the CpG’s mean and CpGs were mean-centered. Batch effects were detected, for instance strong correlation of leading principal components to the bisulfite conversion efficiency (BSCE) in Normal data. To remove such known and additional unknown batch effects we adjusted the data using Surrogate Variable Analysis [39] (bioconductor R-package ‘SVA’), keeping age and phenotype as variables of interest. Using the default method “be”, 5, 5, 4, 2, 4 and 13 surrogate variables were found in Normal, Normal HPV+, Normal HPV-, CIN2+(a), CIN2+(b) and Cancer, respectively. The data were adjusted accordingly. After adjustment no significant correlation of BSCE to leading principal components could be detected. Finally, data were COPA transformed (Cancer Outlier Profile Analysis [40]).

### Feature selection: testing for average methylation differences, methylation variation differences and methylation-age-correlation, corresponding combinations

Traditionally, DNAm analysis tested for differences between the average methylation of case and control samples [33], but different methylation variability [9] and methylation-age-correlation [41] can be additional indicators of risk CpGs [15]. We therefore tested for differential methylation, differential variability and age-correlation of CpGs in the six datasets. It is not a priori clear which test is most appropriate for differential methylation and differential variability in our context (i.e. which test gives the best prediction results), so we used two different tests in each case: differential methylation was quantified by *t*-test and Mann–Whitney *U* test, differential variability by Bartlett’s test and the much less outlier sensitive Levene's test (Brown–Forsythe test gave results between Bartlett’s and Levene’s, but generally close to Levene’s, so was not considered any further). Methylation-age-correlation was tested by comparing the test statistic $r/\sqrt{\left(1-r^{2}\right)/\left(n-2\right)}$ to a t-distribution (*n* = #samples, *r* = Pearson’s correlation coefficient, df = *n*-2). Together, the tests give five (two-sided) p-values per CpG (per dataset). The most significant CpGs, according to a *t*-test for instance, contain both hyper- and hypomethylated sites. The method EVORA [10] uses only hypermethylated and hypervariable CpGs. To also study the prediction performance of MS-SPCA based on pure hyper- or hypo-sites we assign 10 additional p-values (5 ‘hyper-p-values’ and 5 ‘hypo-p-values’) to each CpG, yielding altogether 15 p-values per CpG (per dataset). This is done by the following scheme: the hyper-*t*-test-p-value equals the calculated *t*-test-p-value if the mean methylation of the case samples is larger than the mean methylation of the control samples, otherwise we set hyper-*t*-test-p-value = 1. Equivalently, the hypo-*t*-test-p-value can only be <1 if the mean methylation of the case samples is smaller than the mean methylation of the controls. The ‘hyper-‘and ‘hypo-p-values’ for the other tests were obtained by a similar procedure.

Genome-wide significance was found by calculation of corresponding q-values (measure of false discovery rate [42]), using the R library ‘qvalue’. ‘Hyper-‘and ‘hypo-q-values’ were obtained by only considering the subsets of CpGs with corresponding p-values < 1.

The 15 p-values per CpG allow 15 different (‘simple’) CpG orders for later usage in the supervised PCA (see below).

#### Additional CpG orders by combining ‘simple’ ones – ‘combi’ orders

By combining different ‘simple’ CpG orders we obtain additional ‘combi’ orders. Combining p-values to get new ordered lists is an interesting problem in its own right, a new combination of two p-values was recently suggested [43]. Here we use a simple ranking method to combine any number of lists: first, the ranks of all CpGs in all lists are calculated. Adding of rank-lists produces an equal-weight new rank-list. Ordering according to ascending rank-sums gives a final ‘combi’ order. CpGs with low rank sums are the most significant ones. Instead of just simple summation (equal weights), multiplication with a corresponding weight-vector allows arbitrary weighting of lists. Appropriate weights could for instance correspond to significance ratios (or the corresponding log) of most significant elements. We consider combinations of three test results (a differential methylation test, a differential variability test and the age-correlation test). The relative importance of each test result is expressed by a three-dimensional weight vector. We use the following method to evenly sample the combinations space: (1,0,0) indicates CpG ranking according to a test for average methylation difference (t- or MWU test), (0,1,0) ranking according to a test for methylation variation difference (Bartlett’s or Levene’s test) and (0,0,1) ranking according to methylation-age-correlation. We systematically tested many different weight vectors, ranging from (1,0,0) to (1,2^9^,2^9^) in steps of factor 2, i.e. 277 different vectors (all pairwise cosine distances > 0). Note that a ranking according to for instance the weight vector (1,2^9^,2^9^) yields nearly the same best ranking CpGs as a ranking according to (0,1,1), so this systematic sampling covers the combinations space.

#### Creation of models to be tested in training data cross-validation

For each given weight 30 different numbers of best ranking CpGs were tested (50,100,150,…,1500). Together, we tested 4×277×30 = 33,240 models (4 combinations of the two different tests for average methylation difference and methylation variation difference). The tested CpG-lists contain both hyper- and hypo-sites; for instance CpGs ordered according to a *t*-test contain hyper- and hypo-methylated sites, CpGs ordered according to Levene’s test contain hyper- and hypo-variable sites. We found that the type of test (t or MWU, Bartlett’s or Levene’s) is less important than the parameters weight and number of CpGs tested. We tested additional models taking into account only *t*-test and Bartlett’s test rankings (21 different weights, from 1,0,0 to 1,2^9^,0), for the three cases of CpG lists containing hyper- and hypo-sites, only hyper-sites (i.e. only hypermethylated and hypervariable) and only hypo-sites, together 3×21×30 = 1,890 models. In total 35,130 models were tested for cross-validation performance (in a given training dataset).

### Selection of models performing well in training data cross-validation

In cross-validation, parts of the data are used for training and the complement for testing. Here we used the leave-one-out method (LOO), but 5- or 10-fold cross-validation could be used instead. The most significant CpGs were identifed in the training data (the training part of the training data) and the corresponding first principal component was calculated (training-PC1). Using the same CpGs in the test data (the test part of the training data), test-PC1 was obtained by multiplication of the test data methylation matrix with the leading Eigenvector of the training data covariance matrix. Based on the coordinates of samples on the training-PC1, together with the known phenotype (known in training data), prediction of test data was done according to the coordinates of samples on the test-PC1. Using this method prediction accuracies were obtained for all the 35,130 models tested.

The original SPCA method [12] would use the model performing best in the training data for prediction of independent test data. However, we found that the model with the best cross-validation performance is often not the best model for prediction of independent test data. Using the CIN2+(b) data for training we also found many models with cross-validation prediction accuracy = 1, so this parameter is not sufficient to select one best model.

### Model-selection-supervised principal component analysis (MS-SPCA)

Instead of picking just one model for independent test data prediction Model-Selection-SPCA (MS-SPCA) considers many models performing well in the training data cross-validation and from these selects specific models for final prediction, according to additional parameters obtained from the test data. We use the two parameters *Eval1* and *EV1dist* which carry partly independent information. *Eval1* is the normalized largest eigenvalue of the covariance matrix Cov_test_ (taken from the methylation matrix of the test data), considering only the CpGs from the given model. *EV1dist* is the Euclidean distance between the leading Eigenvectors of Cov_train_ and Cov_test_ (the model’s covariance matrix in the training data and in the test data). The smaller *EV1dist* and the larger *Eval1*, the more likely the model fits well to the test data and makes a good prediction (cf. Figure 1). All models were ordered according to the single parameter *Eval1-EV1dist* (standardized numbers). Figure 2 shows that this ordering correlates well with the prediction performance measure AUC. Finally, to increase the robustness of prediction, we calculated cumulative risk scores for the test samples by adding the risk scores of the first 1,2,3,… models (ordered by *Eval1-EV1dist*). Red curves in Figure 2 show the corresponding AUC. The final prediction results (Tables 6 and 7) correspond to the cumulative risk scores from the first n = 5 models (n = 1 means taking the single best ranking model alone for prediction, any n < 100 gives very similar results).

Mathematica code performing all steps from feature selection to MS-SPCA is provided in the Additional file 3.

### Enrichment analyses

Known cervical cancer genes were taken from the Cervical Cancer Gene Database (CCDB [19]), and developmental genes (PCGTs) from [18]. Enrichment was calculated with a hypergeometric test, using 27,578 and 20,000 as background sample sizes for genome scale CpG- and gene-enrichment analyses, respectively.

## Competing interests

The author declares that he has no competing interests.

## Supplementary Material

Additional file 1 **Contains supporting information. Figure S1.** Performance of the first four principal components in separating cases from controls in the Normal data. **Figure S2.** Models trained on Normal HPV+ data. Two parameters - used to select the final prediction models. **Figure S3.** Models trained on Normal HPV+ data. Performance of prediction (AUC). **Figure S4.** Models trained on Normal HPV+ data. Description of models used for predictions (weights and # CpGs). **Figure S5.** Models trained on Normal HPV- data. Two parameters - used to select the final prediction models. **Figure S6.** Models trained on Normal HPV- data. Performance of prediction (AUC). **Figure S7.** Models trained on Normal HPV- data. Description of models used for predictions (weights and # CpGs). **Figure S8.** Larger test sets allow MS-SPCA to more reliably select the best performing models. **Figure S9.** DNAm patterns can predispose to HPV infection. Click here for file

Additional file 2 Contains detailed information about the 139 most salient CpGs. Click here for file

Additional file 3 Contains Mathematica files used for statistical tests (MS-SPCA-1-GpG-analyses.math), LOO cross-validation (MS-SPCA-2-15lists-forLOO.math, MS-SPCA-3-LOO.math) and independent data prediction (MS-SPCA-4-prediction.nb). Click here for file
