# Supplementary material for: Phenotype prediction based on genome-wide DNA methylation data
Source: BMC Bioinformatics. 2014 Jun 17;15:193. doi: 10.1186/1471-2105-15-193 (PMC4073816; doi:10.1186/1471-2105-15-193)
Supplement: Additional file 1 — Contains supporting information. Figure S1. Performance of the first four principal components in separating cases from controls in the Normal data. Figure S2. Models trained on Normal HPV+ data. Two parameters - used to select the final prediction models. Figure S3. Models trained on Normal HPV+ data. Performance of prediction (AUC). Figure S4. Models trained on Normal HPV+ data. Description of models used for predictions (weights and # CpGs). Figure S5. Models trained on Normal HPV- data. Two parameters - used to select the final prediction models. Figure S6. Models trained on Normal HPV- data. Performance of prediction (AUC). Figure S7. Models trained on Normal HPV- data. Description of models used for predictions (weights and # CpGs). Figure S8. Larger test sets allow MS-SPCA to more reliably select the best performing models. Figure S9. DNAm patterns can predispose to HPV infection. [file 1471-2105-15-193-S1.doc]

**(a)**


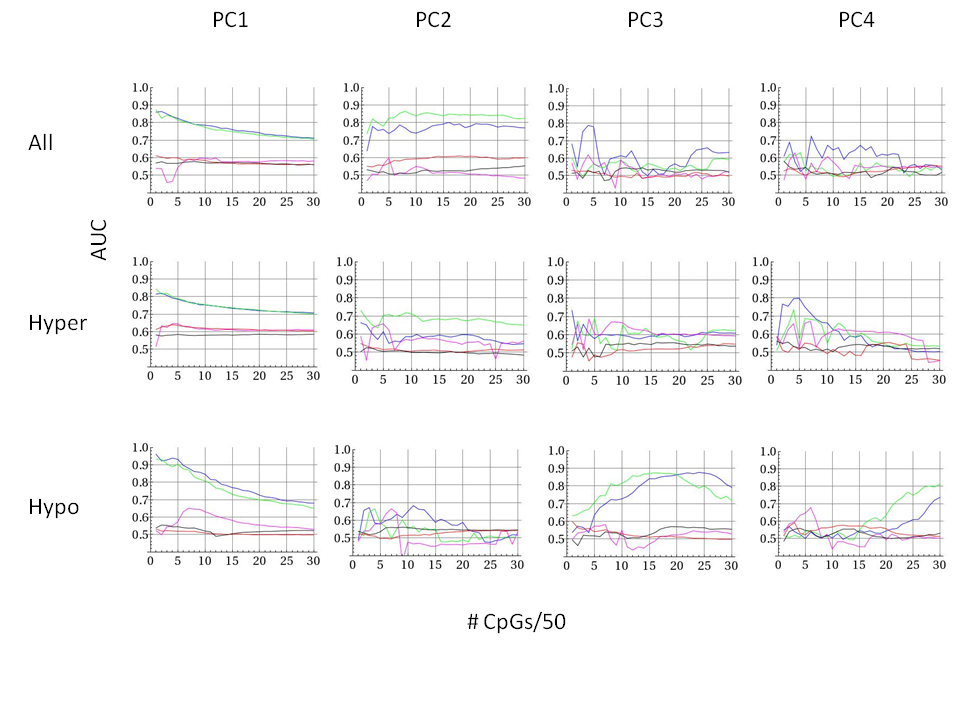


**(b)**


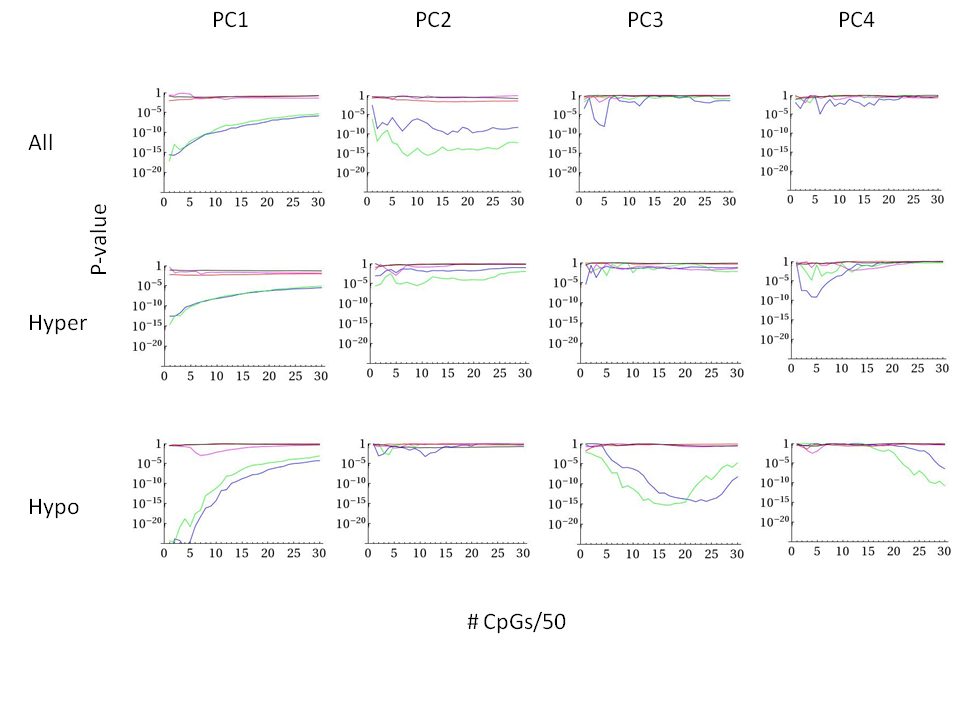


**Fig.S1 Performance of the first four principal components (PCs) in separating cases from controls in the Normal data.**

CpGs were ordered according to five different tests (t-test (blue), Mann-Whitney U test (green), Bartlett’s test (magenta), Levene’s test (red), and test for methylation-age-correlation (black)). The first 50, 100, 150, …, 1500 CpGs were taken and the corresponding first four PCs calculated. These PCs were tested for separation (note that this is no prediction). (a) AUC, (b) corresponding p-values (t-test). PC1 separates well when the first few hundred CpGs ordered according to methylation difference tests are used. For higher numbers of CpGs the importance of PC1 declines and the next PCs are getting more important.

**Prediction of later neoplastic transformations**

Figs. S2-7 show results of phenotype prediction in 5-fold cross-validation of Normal HPV+ (Figs. S2-4) and HPV- samples (Figs. S5-7), as well as corresponding predictions of the independent datasets CIN2+(a), CIN2+(b) and Cancer. 3x30x277=24,930 models were tested in each case in internal LOO cross-validation (same weights and # CpGs as discussed in Methods, considering all CpGs, or only hyper- or hypo-CpGs).

For all HPV+ training data models with LOO-prediction-accuracy>0.62 were taken for test data predictions, for HPV- training data models with LOO-prediction-accuracy>0.65 were taken.


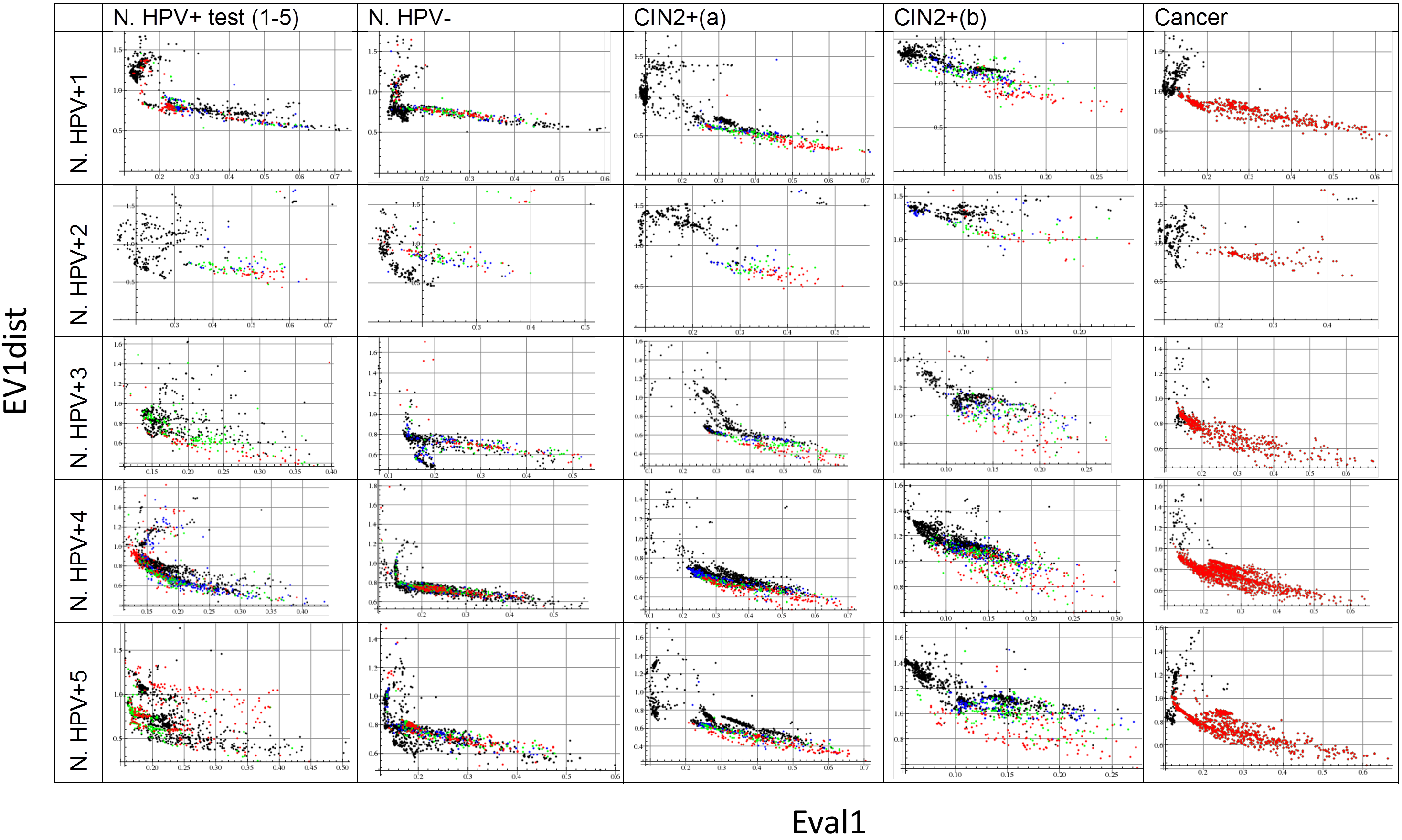


**Fig.S2 Models trained on Normal HPV+ data. Two parameters - used to select the final prediction models.**

Each dot corresponds to one model that performs well in cross-validation in the training data. Each row corresponds to a given training data (name on the left), each column to the corresponding test data (name in header). For better visualization, the 10% of the models predicting the test data best are shown in red, the next 10% (between decile 1 and 2) are coloured green and the next (between decile 2 and 3) blue. Black dots represent the other 70%.


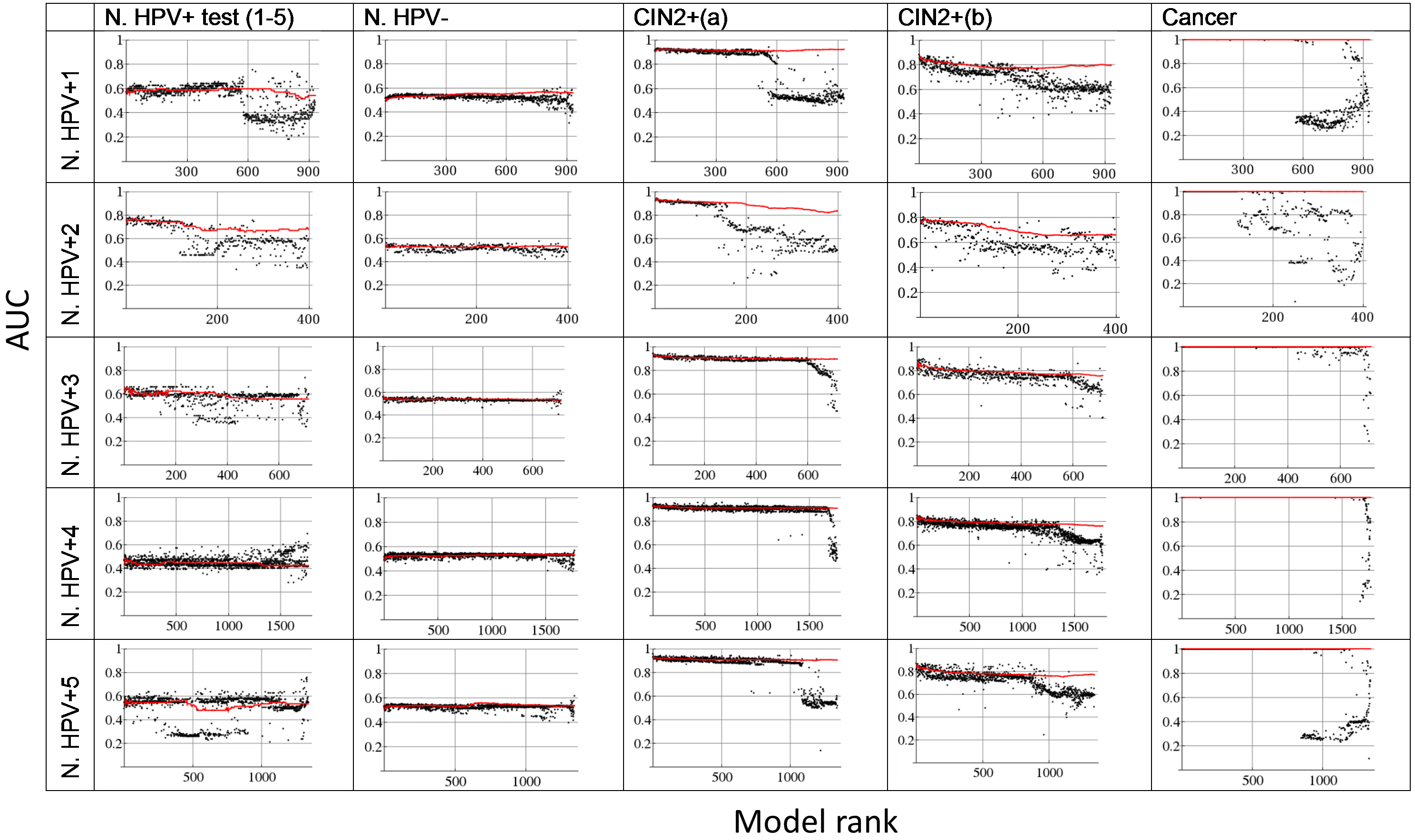


**Fig.S3. Models trained on Normal HPV+ data. Performance of prediction (AUC).**

Each row corresponds to a given training data, each column to a test data, each dot to one model. Models are ordered according to *Eval1*-*EV1dist*, rank 1 corresponds to the model with the highest value. The red line shows the AUC resulting from cumulative risk scores (cf. Methods). The values of the red lines at model rank 5 are given in Tab.7.


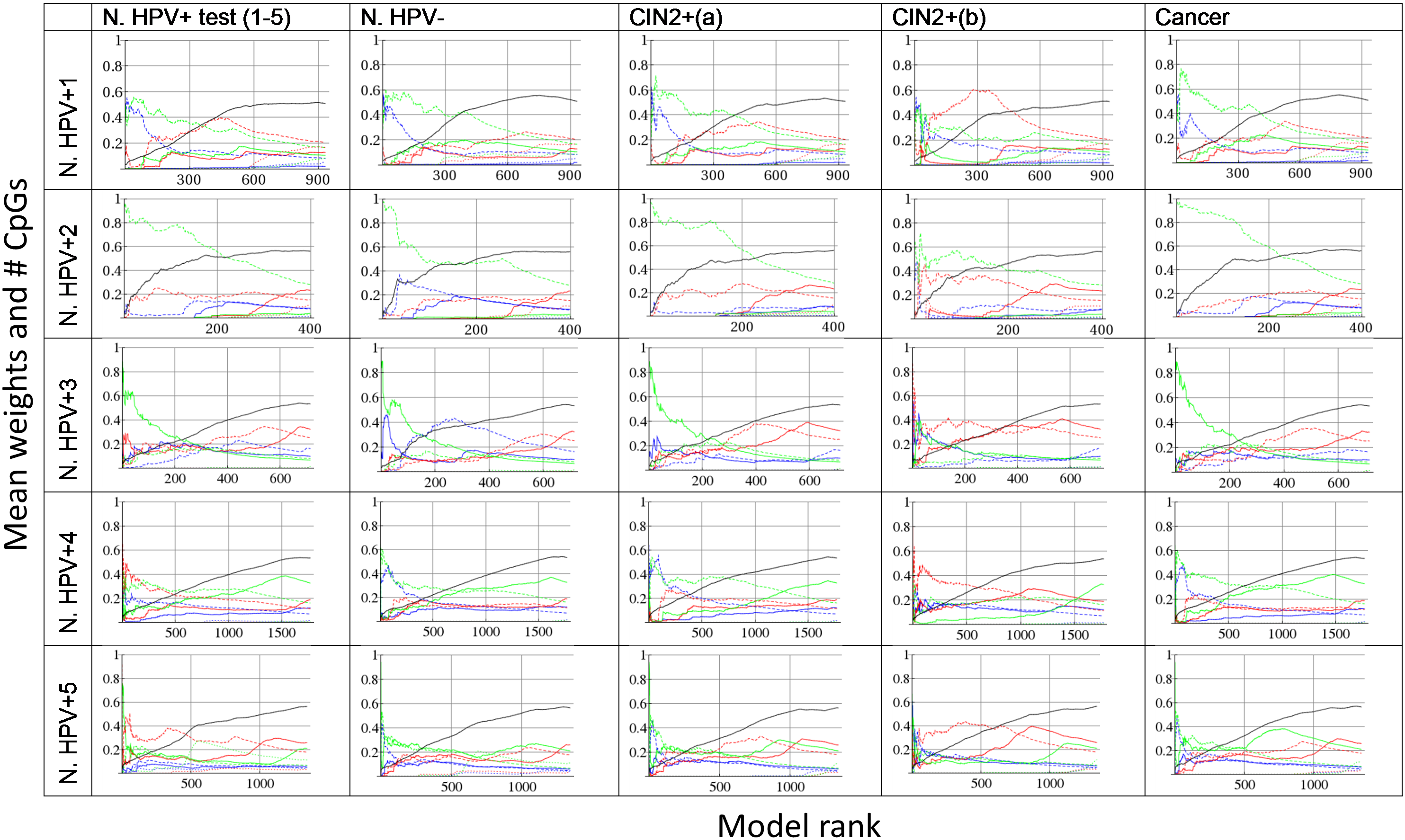


**Fig.S4. Models trained on Normal HPV+ data. Description of models used for predictions (weights and # CpGs).**

Each row corresponds to a given training data, each column to a test data. Models are ordered according to *Eval1*-*EV1dist*, rank 1 corresponds to the model with the highest value. The black line shows the mean number of CpGs used in the models up to the indicated rank, normalized by the maximum number of CpGs considered (1500). The other lines correspond to the average weights used in the models up to the indicated rank. Blue lines correspond to average methylation difference (t- or MWU test), red to methylation variation difference (Bartlett’s or Levene’s test), green to age-correlation. Solid lines indicate models taking into account both, hyper- and hypomethylated CpGs, dashed lines models using only hyper- and dotted lines models using only hypomethylated CpGs.


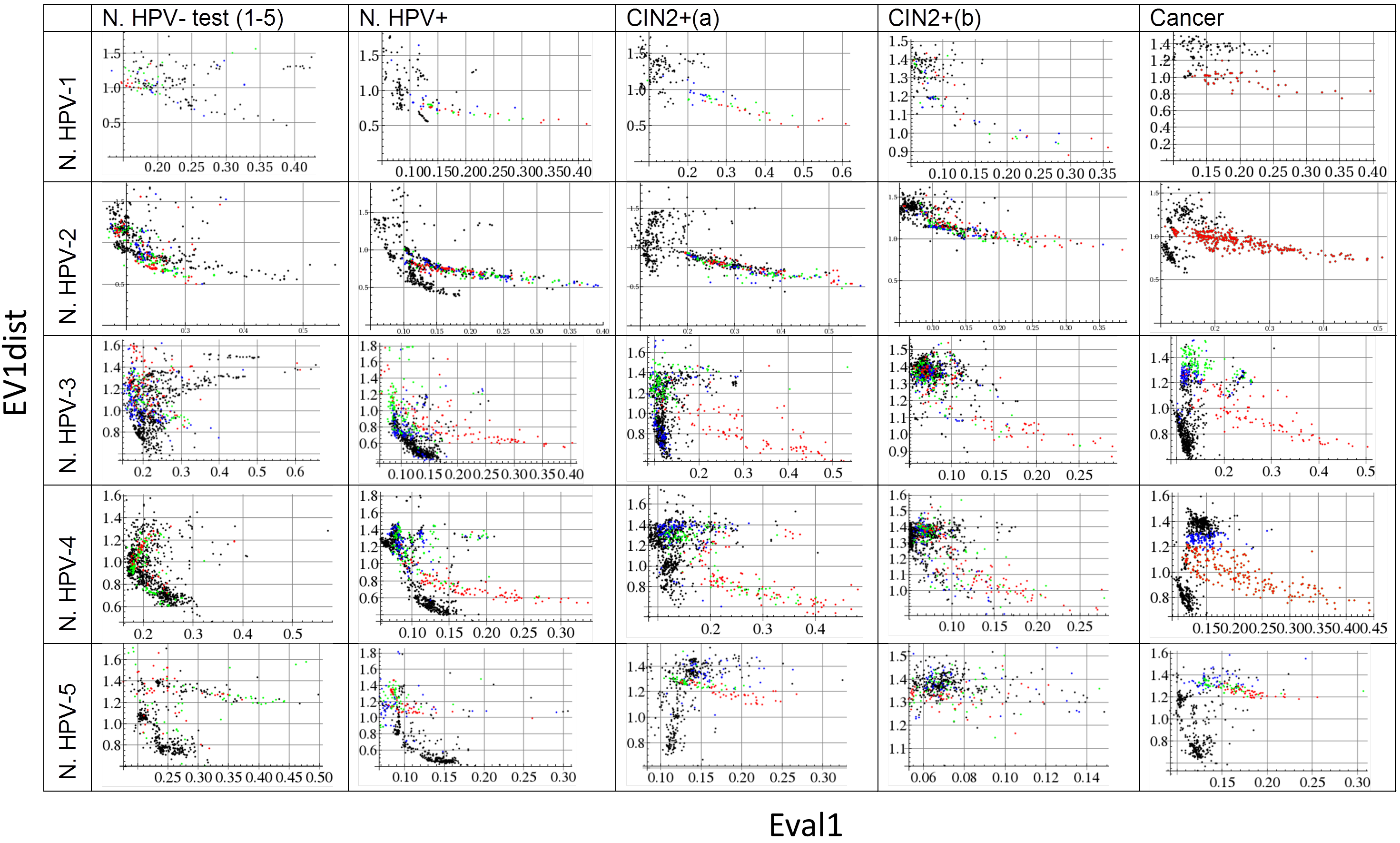


**Fig.S5. Models trained on Normal HPV- data. Two parameters - used to select the final prediction models.**

Each dot corresponds to one model that performs well in cross-validation in the training data. Each row corresponds to a given training data (name on the left), each column to the corresponding test data (name in header). For better visualization, the 10% of the models predicting the test data best are shown in red, the next 10% (between decile 1 and 2) are coloured green and the next (between decile 2 and 3) blue. Black dots represent the other 70%.


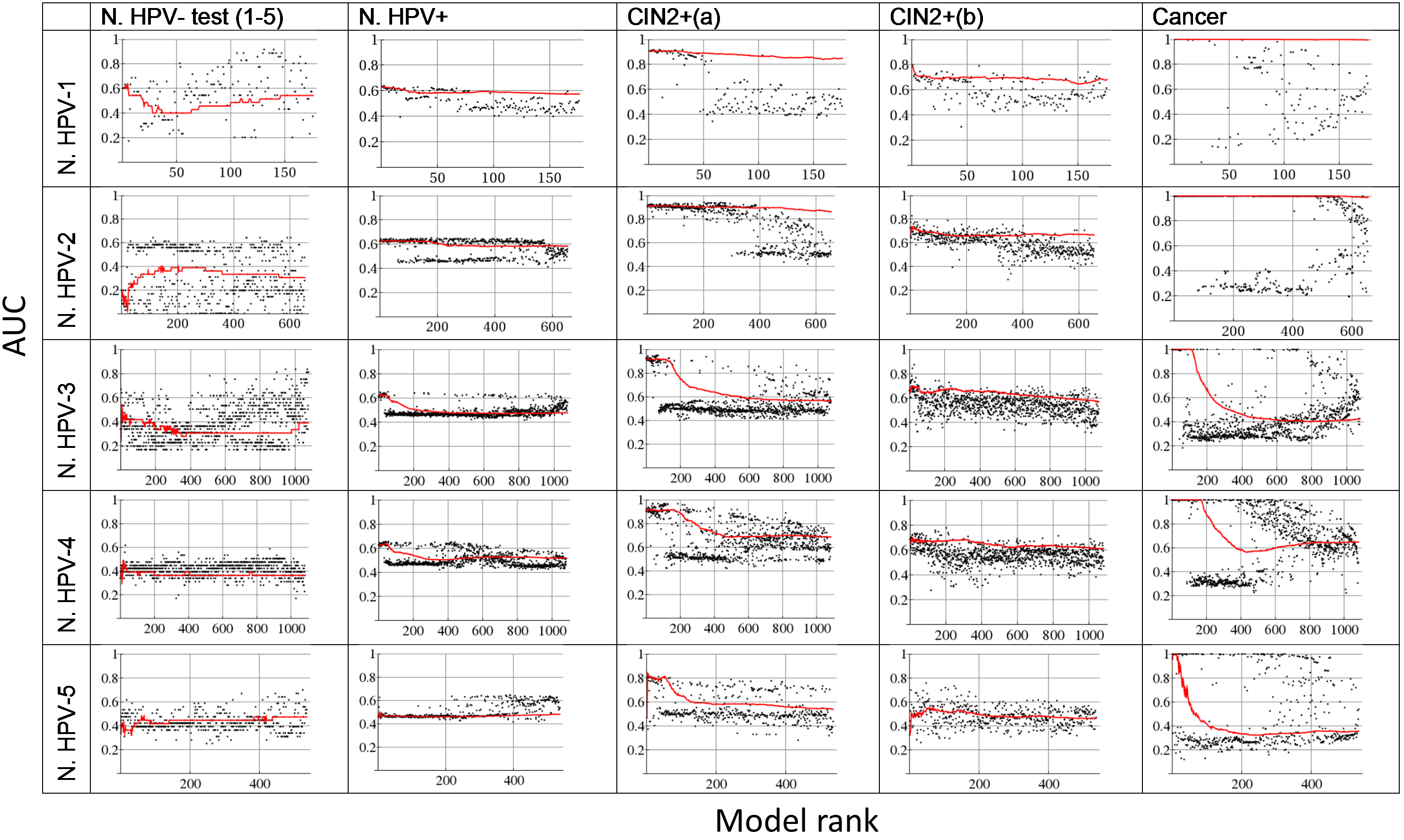


**Fig.S6. Models trained on Normal HPV- data. Performance of prediction (AUC).**

Each row corresponds to a given training data, each column to a test data, each dot to one model. Models are ordered according to *Eval1*-*EV1dist*, rank 1 corresponds to the model with the highest value. The red line shows the AUC resulting from cumulative risk scores (cf. Methods). The values of the red lines at model rank 5 are given in Tab.7.


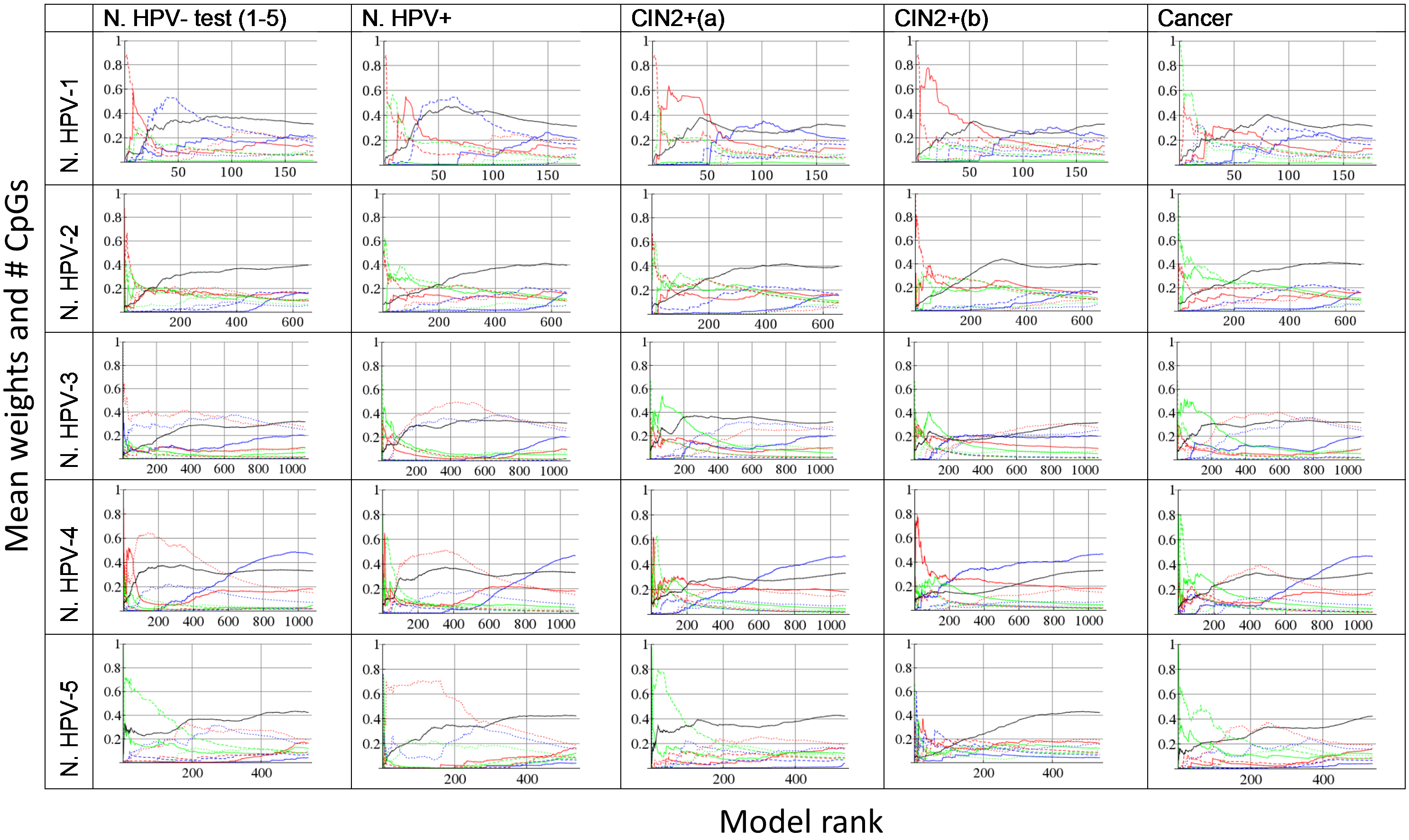


**Fig.S7. Models trained on Normal HPV- data. Description of models used for predictions (weights and # CpGs).**

Each row corresponds to a given training data, each column to a test data. Models are ordered according to *Eval1*-*EV1dist*, rank 1 corresponds to the model with the highest value. The black line shows the mean number of CpGs used in the models up to the indicated rank, normalized by the maximum number of CpGs considered (1500). The other lines correspond to the average weights used in the models up to the indicated rank. Blue lines correspond to average methylation difference (t- or MWU test), red to methylation variation difference (Bartlett’s or Levene’s test), green to age-correlation. Solid lines indicate models taking into account both, hyper- and hypomethylated CpGs, dashed lines models using only hyper- and dotted lines models using only hypomethylated CpGs.


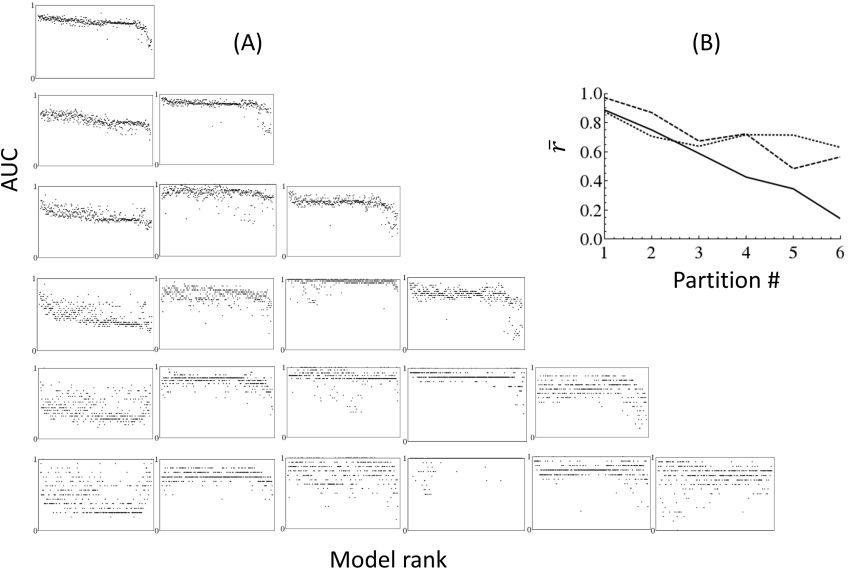


**Fig.S8. Larger test sets allow MS-SPCA to more reliably select the best performing models.**

(A) AUC of models trained on Normal HPV+ data for prediction of the complete CIN2+(b) data (panel 1) and subsets of CIN2+(b) data (partitioned into 2-6 equally sized sets, panels 2-6). (B) Mean Pearson correlation of the AUC of the models ordered according to *Eval1*-*EV1dist* vs. the sorted AUC itself, after partition of the test data [solid CIN2+(b), dashed CIN2+(a), dotted Cancer] into 1-6 subsets.


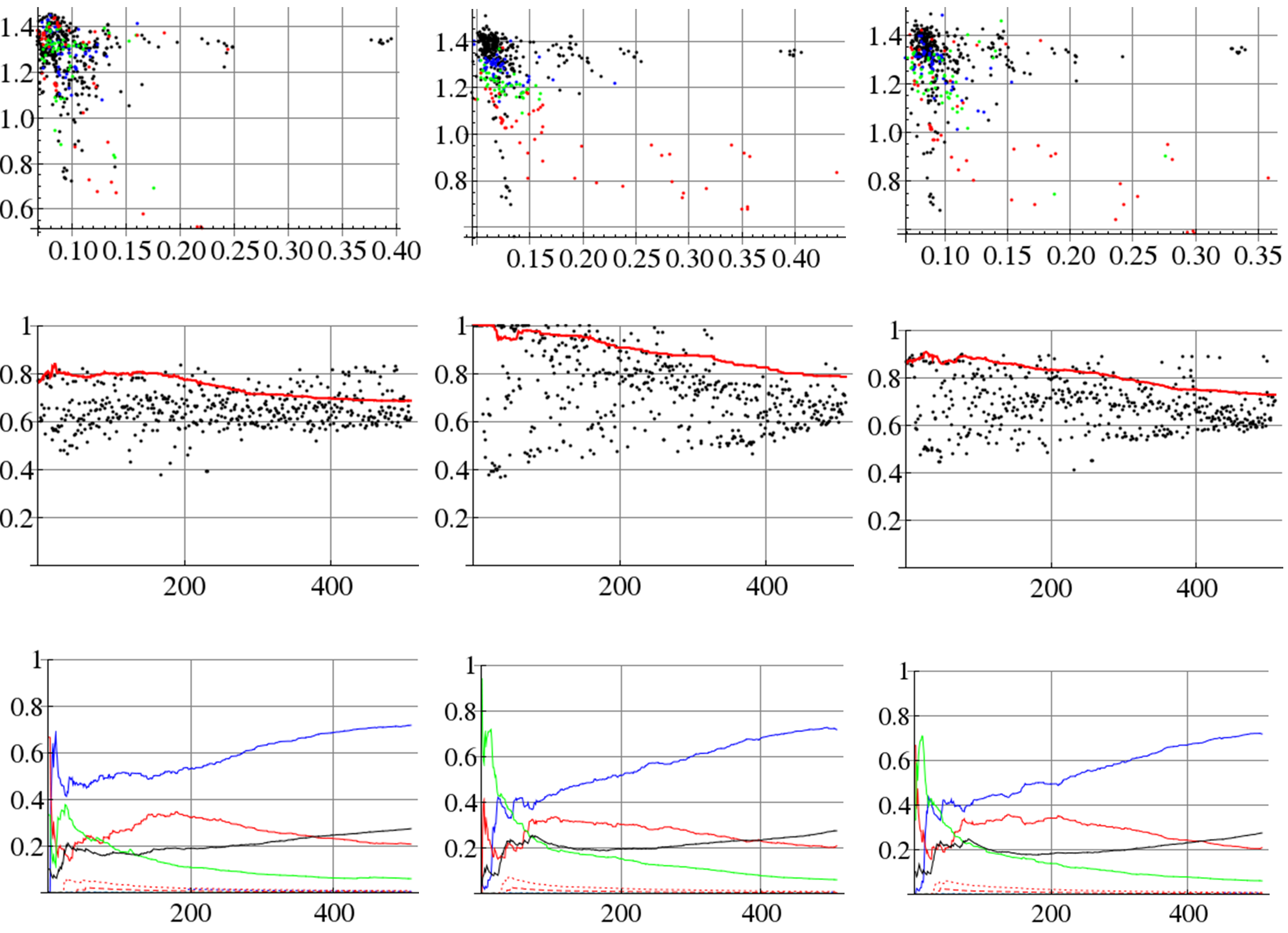


**Fig.S9. DNAm patterns can predispose to HPV infection.**

Using the models trained on Normal HPV- data (all with LOO prediction accuracy>0.65) to predict HPV infection. The three rows correspond to Figs.S2-S4 and S5-S7. Columns 1-3 correspond to 3 different test sets. The cases are identical in all 3 test sets: 28 HPV+ samples where the women remained cytology normal for at least 3 years (taken from the Normal HPV+ data). Controls in column 1 are 14 cytology normal HPV negative samples from the CIN2+(a) data, controls in column 2 are 10 cytology normal samples from the Cancer data (assumed to be HPV-). Controls in column 3 are the 14+10 combined.

Cytology normal HPV- samples in CIN2+(a) and Cancer are generally from older women than the normal HPV+ samples, so we selected older HPV+ samples and younger HPV- samples to approximate age-matching. Cases and controls of the test set in column 1 are age-matched (p~0.2), but in the other columns the controls tend to be older. Note that methylation-age-correlation has the highest weight in the top ranking models in columns 2,3.
